# Tolerability, Safety, and Effectiveness of Two Years of Treatment with Lurasidone in Children and Adolescents with Bipolar Depression

**DOI:** 10.1089/cap.2021.0040

**Published:** 2021-09-17

**Authors:** Melissa P. DelBello, Michael Tocco, Andrei Pikalov, Ling Deng, Robert Goldman

**Affiliations:** ^1^ Division of Bipolar Disorders Research, Department of Psychiatry and Behavioral Neuroscience, University of Cincinnati College of Medicine, Cincinnati, Ohio, USA.; ^2^ Sunovion Pharmaceuticals, Inc., Marlborough, Massachusetts, USA.; ^3^ Sunovion Pharmaceuticals, Inc., Fort Lee, New Jersey, USA.

**Keywords:** second-generation antipsychotic, lurasidone, bipolar disorder, depressive disorder, children, adolescents

## Abstract

***Objectives:*** To evaluate long-term safety and effectiveness of lurasidone in children and adolescents with bipolar depression.

***Methods:*** Participants, ages 10–17 years, with bipolar depression, who completed 6 weeks of double-blind (DB) treatment with lurasidone or placebo were enrolled in a 2-year, open-label (OL) extension study of lurasidone (20–80 mg/d). The primary effectiveness measure was the Children's Depression Rating Scale, Revised (CDRS-R).

***Results:*** A total of 306 participants entered the 2-year extension study; 195 (63.7%) completed 52 weeks, and 168 (54.9%) completed 104 weeks of treatment. For all participants entering the extension study, mean change in CDRS from OL baseline was −13.4 at week 52, and −16.4 at week 104 (−11.3 at last observation carried forward [LOCF]-endpoint). Overall, 31 participants (10.1%) discontinued due to an adverse event (AE); the three most common AEs were headache (23.9%), nausea (16.4%), and somnolence (9.8%). OL treatment with lurasidone was associated with few effects on metabolic parameters or prolactin. Mean change from DB baseline in weight was +4.25 kg at week 52 (vs. an expected weight gain of +3.76 kg), and +6.75 kg at week 104 (vs. an expected weight gain of +6.67 kg), based on the sex- and age-matched United States Center for Disease Control normative data.

***Conclusions:*** For youth with bipolar depression, up to 2 years of treatment with lurasidone was generally well tolerated, safe, and effective with relatively low rates of discontinuation due to AEs, minimal effects on weight, metabolic parameters or prolactin, and continued improvement in depressive symptoms.

Clinical Trial Registration number: NCT01914393.

## Introduction

Up to two-thirds of individuals with bipolar disorder have their onset during childhood or adolescence (Perlis et al. 2004) Results of a meta-analysis of children and adolescents (*N* = 56,103) found a weighted average prevalence of 3.9% for the full spectrum of bipolar disorders in youth (bipolar I, II, and not otherwise specified), and a prevalence of 0.6% for bipolar I disorder (Van Meter et al. 2019). Childhood onset of bipolar disorder is associated with a more chronic and severe course of illness compared with later onset (Birmaher et al. 2006, 2014; Perlis et al. 2009; Lee et al. 2020; Post et al. 2020). In a long-term (mean, 7.7 years) prospective follow-up study, 76% of children were euthymic for less than 50% of the follow-up period (Lee et al. 2020). In addition to the pervasiveness of abnormal mood states, bipolar disorder in children and adolescents is associated with significant impairment in functioning, cognition, and quality of life (Andreou et al. 2013; Bourne et al. 2013; Ratheesh et al. 2013; Martino et al. 2014; Szmulewicz et al. 2017; Lima et al. 2018).

Due to the chronic and recurrent course of bipolar disorder in children and adolescents, long-term pharmacotherapy is typically required, making safety and tolerability crucial factors when determining the choice medications for this vulnerable population. Youth with bipolar disorder have significantly higher rates of obesity, dyslipidemia, insulin resistance, and metabolic syndrome than adolescents without bipolar disorder in the community (Goldstein et al. 2015). As the American Heart Association has noted in a scientific statement: “…bipolar disorder predispose(s) youth to accelerated atherosclerosis and early cardiovascular disease” (see also the “Call to Action” of the Vascular Task Force of the International Society for Bipolar Disorders) (Goldstein et al. 2020). Consequently, the longitudinal course of bipolar disorder is associated with a significantly increased risk of vascular morbidity and mortality resulting in an average reduction in life expectancy of 10–15 years (Westman et al. 2013).

Lurasidone is a second-generation antipsychotic agent with high affinity for D_2_, 5-HT_2A_, and 5-HT_7_ receptors (Ki = 1.0, 0.5, and 0.495 nmol/L, respectively) (Ishibashi et al. 2010). Lurasidone is approved by the U.S. Food and Drug Administration (FDA) for the treatment of bipolar depression in adults as monotherapy, and as adjunctive therapy with lithium or valproate (Loebel et al. 2014a,b). Lurasidone has demonstrated efficacy in the treatment of children and adolescents with bipolar depression (DelBello et al. 2017) and has been approved by the FDA as monotherapy for bipolar depression in participants ages 10–17 years. The antidepressant mechanism of action of lurasidone is not well understood. Interestingly, in standard preclinical models of depression, loss of antidepressant efficacy is observed for 5-HT_7_ knockout mice, suggesting that the antidepressant efficacy of lurasidone may be mediated, in part, by antagonist activity at the 5-HT_7_ receptor (Cates et al. 2013).

Minimal prospective data are available on the safety, tolerability, and effectiveness of antipsychotic agents for the treatment of bipolar depression in youth (quetiapine and olanzapine/fluoxetine combination) (DelBello et al. 2009; Findling et al. 2014; Detke et al. 2015), especially based on long-term studies. In a previously reported double-blind (DB), placebo-controlled, flexible-dose, 6-week trial, lurasidone demonstrated significant efficacy in treating children and adolescents with an acute episode of bipolar depression and was found to be safe and generally well tolerated in a dose range of 20–80 mg/d (DelBello et al. 2017). We report in this study, the results of the 2-year, open-label (OL) follow-up of that study, designed to evaluate the long-term tolerability, safety, and effectiveness of lurasidone in children and adolescents with bipolar depression.

## Methods

This was a 104-week, OL extension study (clinicaltrials.gov) that enrolled participants 10–17 years of age who completed an initial 6-week, DB, placebo-controlled trial evaluating the efficacy and safety of flexible does of lurasidone (20 to 80 mg/d) for the treatment of bipolar depression (NCT02046369) (DelBello et al. 2017).

The study was conducted from November 2013 to October 2018 at 62 centers in 11 countries (Bulgaria, Columbia, France, Hungary, Korea, Mexico, Philippines, Poland, Russia, Ukraine, and United States). The study was approved by an Institutional Review Board/Ethics Committee at each investigational site and was conducted in accordance with the International Conference on Harmonization Good Clinical Practice guidelines and with the ethical principles of the Declaration of Helsinki. The study was monitored by an independent Data and Safety Monitoring Board. After a full explanation of the study was provided, written informed consent was obtained from a parent or legal guardian, and assent was obtained from each participant.

### Participants and study design

Entry into the preceding acute treatment study (DelBello et al. 2017) was limited to male and female participants 10 to 17 years old with a DSM-5 diagnosis of bipolar I disorder (American Psychiatric Association 2013) who were experiencing a major depressive episode with a duration of 1 to 12 months and had a Children's Depression Rating Scale, Revised (CDRS-R) (Poznanski et al. 1996) total score of at least 45 at screening and baseline, and a Young Mania Rating Scale (YMRS) score ≤15 with a YMRS item 1 (elevated mood) score ≤2 at screening and baseline (Young et al. 1978).

Participants were excluded if they met DSM-5 criteria for a current or lifetime diagnosis of schizophrenia or any psychotic disorder, substance use disorder within the past 6 months (except caffeine or tobacco), intellectual disability, or autism spectrum disorder. Participants also were excluded if any clinically significant neurologic, endocrine, or other medical disorder was present at screening that might pose a risk to participants in the study or that might confound interpretation of study results.

Participants were included in the current extension study if they were judged by the site investigator to be suitable for participation in a 104-week OL study and were able to comply with the protocol; if they were not considered by the investigator to be at imminent risk of suicide or injury to self or others; if they exhibited no evidence of moderate or severe extrapyramidal symptoms, dystonia, tardive dyskinesia, or any other movement disorder; and if they were willing to use medically appropriate contraception if sexually active.

To maintain the blind in the preceding acute treatment trial, participants enrolled in the current extension study were started on a dose of 40 mg/d for 1 week, regardless of their treatment group in the original DB study. The dose of lurasidone was adjusted at weekly intervals, in the flexible dose range of 20–80 mg/d, to optimize efficacy and tolerability. Lurasidone was taken orally, once-daily in the evening within 30 minutes after eating.

### Concomitant medication

Concomitant treatment with benzodiazepines, antidepressants, and stimulants for attention deficit/hyperactivity disorder (ADHD) was permitted; treatment with benztropine (≤6 mg/d), or equivalent medications, was permitted as needed for movement disorders; and treatment with propranolol (≤120 mg/d) was permitted as needed for akathisia. Prophylactic use of medications to treat movement disorders was not permitted. Concomitant use of lorazepam, or equivalent benzodiazepine, was permitted at the discretion of the investigator (≤6 mg/d or equivalent dose) for intolerable anxiety/agitation. Benzodiazepine and nonbenzodiazepine sedative/hypnotic agents were also permitted on an as-needed basis for insomnia.

For participants who were hospitalized at the conclusion of the original DB study, continued hospitalization for up to 14 days in the current extension study was permitted. Participants that could not be transitioned to an outpatient setting within 14 days were discontinued from the study.

### Tolerability and safety assessments

Tolerability and safety assessments were performed at each study visit, which occurred every 2 weeks for the first 8 weeks, and every 4 weeks thereafter, until the end of the study.

The presence and severity of spontaneously reported adverse events (AEs) were recorded at each study visit. AE reporting was supplemented by administration of the Udvalg for Kliniske Undersogelser (UKU) Side Effect Rating Scale, a clinician-rated scale consisting of 48 adverse effect items (each rated on a 0–3-point scale, 0-no side effects, 1-mild, 2-moderate, 3-severe) divided into four categories (psychic [0 to 30], neurologic [0 to 24], autonomic [0 to 33], and other [0 to 48]) (Lingjaerde et al. 1987). Mean severity scores were calculated for each side effect category and for the total score. Movement disorders were assessed by the Simpson-Angus Scale, the Barnes Akathisia Rating Scale, and the Abnormal Involuntary Movement Scale. The Columbia Suicide Severity Rating Scale (C-SSRS) Posner et al. 2011) was used to assess suicidal ideation and behavior. Additional safety evaluations included vital signs, height, weight, body mass index (BMI), laboratory tests (metabolic, hormonal, blood chemistries, hematologic parameters), 12-lead electrocardiogram (ECG), Tanner staging, and physical examination. Analysis of change in body weight during OL treatment was performed relative to DB baseline and was compared with sex- and age-adjusted U.S. Centers for Disease Control and Prevention (CDC) normative data (it should be noted that after 6 weeks of DB treatment, participants randomized to lurasidone showed no clinically meaningful difference in weight gain compared with participants randomized to placebo).

### Effectiveness assessments

Effectiveness assessments were performed by qualified site-based raters. In the current OL study, effectiveness measures were considered secondary outcomes and consisted of the following: the CDRS-R (Poznanski et al. 1996), the Clinical Global Impression-Bipolar Version, Severity of Illness (CGI-BP-S) depression score, the Clinician-rated Children's Global Assessment Scale (CGAS) score (Shaffer et al. 1983), the YMRS, the Pediatric Anxiety Rating Scale (PARS 2002), and the Pediatric Quality of Life Enjoyment and Satisfaction Questionnaire (PQ-LES-Q) score (percent of maximum; Endicott et al. 2006). The PQ-LES-Q is a quality-of-life measure and scores within 10% of normative community mean score is typically considered to represent a return to normal levels of quality of life. Note that the YMRS was used as a safety measure in the initial DB study, but was used as an effectiveness measure in the current study, including as one of the composite criteria in the definition of remission.

### Statistical analysis

The safety population consisted of all participants who completed the DB, acute-phase trial, continued into the current extension study, and received at least one dose of OL lurasidone. All tolerability, safety, and effectiveness summaries were based on the safety population. No inferential statistics were calculated because of the absence of a parallel placebo active comparator treatment group. For continuous variables (including both safety and effectiveness variables), descriptive summary statistics (*N*, mean, median, 95% confidence interval [CI]) were reported at DB baseline, OL baseline, each post-OL visit, week-52 endpoint, and endpoint in the OL study. In addition, changes from DB baseline and OL baseline were also reported in a similar way using summary statistics as described above. For treatment-emergent AEs, number and percentage of subjects with one or more events were summarized for overall incidence, serious AEs (SAEs), and discontinuations due to AEs. To analyze the effects of lurasidone on growth parameters, age, and sex-specific z-scores for height and BMI were reported using the World Health Organization growth charts (WHO Study Group. 2006), and age and sex-specific z-scores for body weight were reported using the CDC 2000 growth charts (Ogden et al. 2002). In this OL extension study, to better interpret growth changes in the absence of a placebo-controlled group, expected value of weight per CDC growth reference and expected value of height and BMI per WHO growth chart were derived for each subject at study visits; mean expected changes relative to DB baseline were summarized for these growth parameters.

The CDRS-R total score was the primary, prespecified efficacy measure in the initial DB, placebo-controlled trial, and it was the primary effectiveness measure in the current extension study. Secondary effectiveness measures were the CGI-BP-S depression score, the PARS total score, the CGAS total score, and the PQ-LES-Q score. Descriptive statistics for these effectiveness measures were analyzed separately. Treatment response was defined as ≥50% reduction in the CDRS-R total score from baseline (either DB baseline or OL baseline), calculated based on both observed case data, and last observation carried forward (LOCF) data. The number and proportion of responders relative to baseline by study visit were summarized. In a *post-hoc* analysis, the following criteria were used to define remission: a CDRS-R total score ≤28, a YMRS total score ≤8, and a CGI-BP-S depression score ≤3. A YMRS total score ≤12 is the standard criterion for mania symptom remission in bipolar mania clinical trials, however a more stringent YMRS criterion was used in the current study with an index episode of depression. Treatment-emergent mania was defined *a priori* as a YMRS total score ≥20 for 2 consecutive visits (or at one visit, if it was the participant's final visit), or any treatment-emergent AE of mania or hypomania.

## Results

**FIG. 1.**
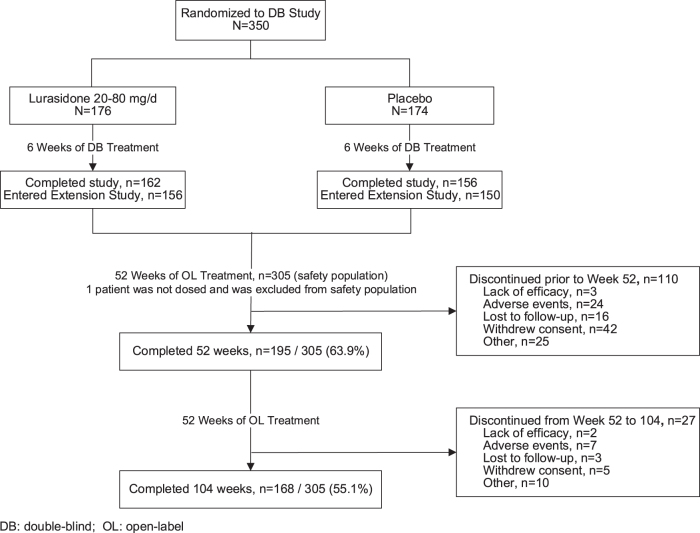
Participant disposition.

A combined total of 318 participants completed the 6-week, DB, placebo-controlled trial, of whom 306 participants (96.2%) provided informed consent/assent and continued into the current OL extension study, including 156 participants initially randomized to lurasidone and 150 participants initially randomized to placebo (Fig. 1). Among participants entering the OL extension study who received a dose of study medication (safety population), 153/305 (50.2%) were male and 152 (49.8%) were female; mean (standard deviation [SD]) age was 14.4 (2.2) years; and 235 (77.0%) were White, 26 (8.5%) were Black, 10 (3.3%) were Asian, and 34 (11.1%) were other races. A total of 27.9% reported a previous hospitalization for bipolar disorder and 61.3% had previously been treated with an antipsychotic medication before DB treatment phase. At OL baseline, participants previously treated with lurasidone and placebo in the initial DB phase reported mean (SD) CDRS-R score of 36.6 (12.5) and 41.9 (13.8), respectively, and mean (SD) CGI-BP-S depression scores of 3.0 (1.1) and 3.4 (1.1), respectively.

In the extension study, 195 (63.7%) of participants completed 52 weeks, and 168 (54.9%) completed 104 weeks of treatment (Fig. 1). Reasons for discontinuation consisted of withdrawal of consent (15.4%), AE (10.1%), lost to follow-up (6.2%), protocol violation (6.2%), lack of efficacy (1.6%), and other reasons (5.2%; Fig. 1).

The mean (SD) daily dose of lurasidone averaged over the 104-week OL treatment period was 52.1 (15.7) mg/d; and the modal dose of lurasidone utilized by participants during OL treatment was 20 mg (6.6% of participants), 40 mg (41.6%), 60 mg (25.9%), and 80 mg (25.9%). Mean daily dose of lurasidone across the 104-week OL treatment is similar between the 10–12-year-old group and the ≥13-year-old group (52.8 mg vs. 54.9 mg). Pill counts indicated that compliance was high, with only two participants noncompliant (defined as use of <75% or >125% of prescribed study medication) on three or more visits during 104 weeks of study treatment.

Concomitant medication included benzodiazepines (16.7%), antidepressants (3.6%), anticholinergic medication (5.2%), and stimulant or treatment of ADHD (15.4%). At least one dose of a mood stabilizer (a protocol deviation) was taken by eight participants (2.6%) during the 104-week treatment period.

### Tolerability and safety

AEs with an incidence ≥5% are summarized in Table 1. Overall, 41/305 participants (13.4%) experienced an AE that was rated as severe, and 31/305 participants (10.2%) discontinued due to an AE. Among participants reporting a treatment-emergent AE, 152/252 (60.3%) had resolved before the end of the study, 25/252 (9.9%) were less severe, and 74 (29.4%) were ongoing (outcome for one participant was unknown). The incidence of individual AEs was similar (<5% difference), regardless of initial DB treatment assignment (lurasidone or placebo). A total of 6.9% of participants (*n* = 21) reported an extrapyramidal symptom (EPS)-related treatment-emergent AE (non-akathisia; Table 1).

**Table 1.** Treatment-Emergent Adverse Events (≥5%; Safety Population)

AEs, %	Lurasidone 20–80 mg (*N* = 305)
Headache	23.9
Nausea	16.4
Somnolence	9.8
Weight increased	9.5
Anxiety	8.5
Nasopharyngitis	8.5
Vomiting	8.2
Insomnia	7.2
Extrapyramidal events^a^	6.9
Akathisia	6.2
Rhinitis	6.2
Fatigue	5.9
Diarrhea	5.9
Any AE	82.6

^a^ Parkinsonism, dyskinesia, dystonia, extrapyramidal disorder, hypokinesia, salivary hypersecretion, tardive dyskinesia, torticollis, or psychomotor hyperactivity.

AE, adverse event.

The following AEs led to study discontinuation in two or more participants, with some discontinuing participants reporting more than one event: akathisia (*n* = 3), bipolar I disorder (*n* = 3), depression (*n* = 4), mania (*n* = 3), suicidal ideation (*n* = 5), and suicide attempt (*n* = 6). Most of these AEs were categorized as SAEs (see below).

A total of 37 participants had a SAE, with the majority assessed by the investigator as not related to study drug. SAEs reported by one participant each were abdominal pain, bezoar, osteomyelitis, accidental overdose, contusion, fractures of the clavicle, tibia, and wrist, frostbite, intentional overdose, benign ovarian tumor, convulsion, emotional disorder, intentional drug misuse, suicidal behavior, and nephrolithiasis. SAEs reported by two participants each were appendicitis and hand fracture. SAEs reported by three or more participants each were bipolar disorder or bipolar I disorder (*n* = 10), depression (*n* = 3), suicidal ideation (*n* = 5), and suicide attempt (*n* = 6). There were no deaths in the study.

A similar proportion of participants in the 10–12 vs. ≥13-year age groups reported at least one AE (82.3% vs. 82.7%), however, the proportion was higher in the younger age group for study discontinuation due to an AE (19.4% vs. 7.8%) and rate of SAEs (17.7% vs. 10.7%).

The UKU severity scores at DB and OL baselines, weeks 52, 104, and LOCF-endpoint, respectively, showed a gradual improvement (reduction in severity) over time in total side effect score (7.16, 3.78, 1.81, 1.29, 2.03) for the psychic side effects score (5.98, 2.94, 1.24, 0.86, 1.37), neurological side effects score (0.17, 0.13, 0.11, 0.02, 0.07), autonomic side effects score (0.35, 0.28, 0.16, 0.18, 0.21), and other side effects score (0.67, 0.43, 0.31, 0.23, 0.38).

On the C-SSRS, the proportion of participants with treatment-emergent or worsening suicidal ideation (relative to DB baseline) was 12.5% (*n* = 38), and the proportion with emergent or worsening suicidal behavior was 3.0% (*n* = 9).

For the overall safety population (*N* = 305) mean changes from DB baseline to weeks 52 and 104, respectively, were small and not clinically meaningful for the Simpson Angus Scale 10-item mean score (both change scores were less than 0.01), the Barnes Akathisia Scale total score (+0.0 and 0.0), and the Abnormal Involuntary Movement Scale total score (0.0 and +0.01).

**FIG. 2.**
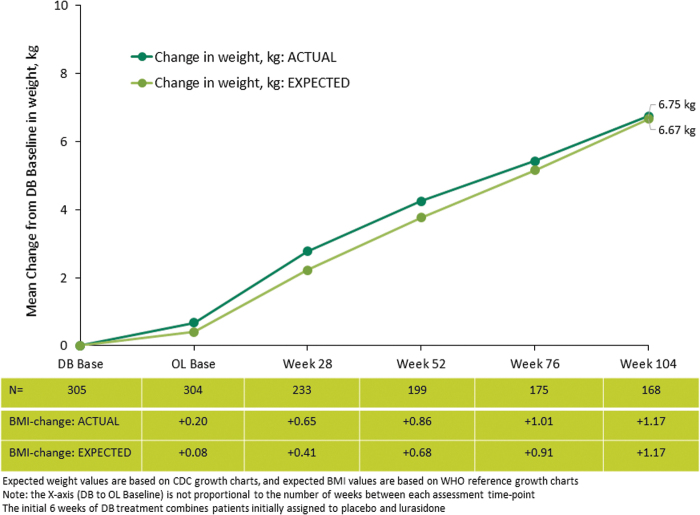
Change from DB baseline in weight and BMI: actual versus expected. BMI, body mass index; DB, double-blind.

For the overall safety population (*N* = 305) mean changes from DB baseline in actual weight (kg) during 104 weeks of OL treatment with lurasidone was similar to the expected change in weight based on CDC growth charts (Fig. 2) (Ogden et al. 2002). Mean change in BMI (kg/m^2^) was also very similar to the expected change in BMI based on WHO growth charts (Fig. 2). The proportion of participants with normal weight (i.e., BMI between 5th and 85th percentile based on WHO reference values) was 62.3% at DB baseline, and this proportion increased at week 52 (70.4%) and week 104 (74.4%).

Treatment with lurasidone was associated with small median changes from DB baseline at both weeks 52 and 104 in total, high-density lipoprotein, low-density lipoprotein, triglycerides, glucose, hemoglobin A1C, and insulin (Table 2); median changes in prolactin were minimal (≤1.5 ng/mL) in both male and female participants (Table 2).

**Table 2.** Change in Laboratory Parameters from Double-Blind Baseline (Safety Population)

	Lurasidone 20–80 mg	
	Week 52 (*N* = 197)	Week 104 (*N* = 162)
Total cholesterol (fasting), median change, mg/dL	0.0	−3.0
HDL cholesterol (fasting), median change, mg/dL	−1.0	−2.0
LDL cholesterol (fasting), median change, mg/dL	−2.0	−3.0
Triglycerides (fasting), median change, mg/dL	+1.0	+8.0
Glucose (fasting), median change, mg/dL	+1.0	+1.0
Hemoglobin A1C, mean change, %	0.0	0.0
Insulin, mean change, mU/L	+0.30	+0.30
Prolactin, median change, ng/mL		
Female	+0.9	+1.1
Male	+1.1	+1.5

HDL, high-density lipoprotein; LDL, low-density lipoprotein.

During OL treatment with lurasidone, no clinically meaningful changes were observed in heart rate, ECG, orthostatic blood pressure (systolic or diastolic), respiratory rate, or body temperature. On serial ECG assessments during up to 104 weeks of OL treatment, no participant had a QT interval corrected for heart rate using Fridericia's formula (QTcF) value ≥460 ms, and one participant had an increase from OL baseline in QTcF that was ≥60 ms (but with a maximum QTcF <460 ms).

### Effectiveness

**FIG. 3.**
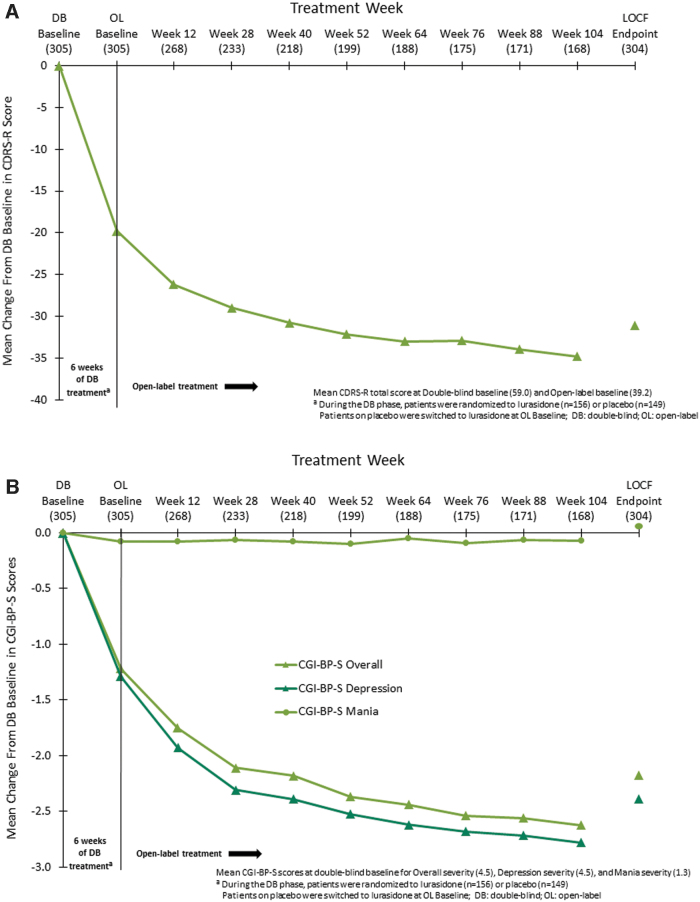
Change from DB baseline in severity scores (OC analyses) **(A)**. CDRS-R total score **(B)**. CGI-BP-S depression score. CDRS-R, Children's Depression Rating Scale, Revised; CGI-BP-S, Clinical Global Impression-Bipolar Version, Severity; DB, double-blind; OC, observed case analysis.

For all participants entering the extension study, mean week 6 change in CDRS-R total score from DB baseline to OL baseline was −19.8, resulting in a CDRS-R total score of 39.2 at OL baseline. At OL baseline, the placebo group was switched to lurasidone. After 6 weeks of OL treatment with lurasidone, mean change from DB baseline in CDRS-R total score was comparable for both the initial (DB phase) lurasidone and placebo treated participants (−25.6 vs. −23.0). For all participants, continued improvement from DB baseline was observed for CDRS-R total score during up to 104 weeks of treatment with OL lurasidone (Fig. 3**A**). The mean change from OL baseline in the CDRS-R total score was −13.4 at week 52, and −16.4 at week 104 (−11.3 at LOCF-endpoint). To verify the impact of early dropouts on change in CDRS-R total score, two sensitivity analyses were conducted to explore the robustness of change from DB baseline and change from OL baseline in CDRS-R total score. The results of these sensitivity analyses (summarized in the online Supplementary Appendix SA) confirmed the effectiveness of lurasidone therapy in terms of change in CDRS-R total score.

Continued improvement from DB baseline was also observed for both the CGI-BP-S depression and CGI-BP-S overall scores (Fig. 3B); no change was observed on the CGI-BP-S mania score. At OL baseline, the mean (SD) YMRS total score (considered a safety outcome in the original protocol) was 5.1 (3.4). The mean (SD) change in YMRS total score was −1.0 (3.4) at week 52, −1.4 (3.7) at week 104, and −0.4 (4.7) at LOCF-endpoint. Treatment-emergent mania occurred in four participants (1.3%) during the 104 weeks of OL treatment with lurasidone.

A small improvement in mean (±SD) PARS total scores from DB baseline (mean = 11.1) was observed over time, with a reduction of -6.7 ± 6.9 at week 52 and -8.4 ± 7.4 at week 104 (-6.4 ± 8.0 at LOCF-endpoint).

**FIG. 4.**
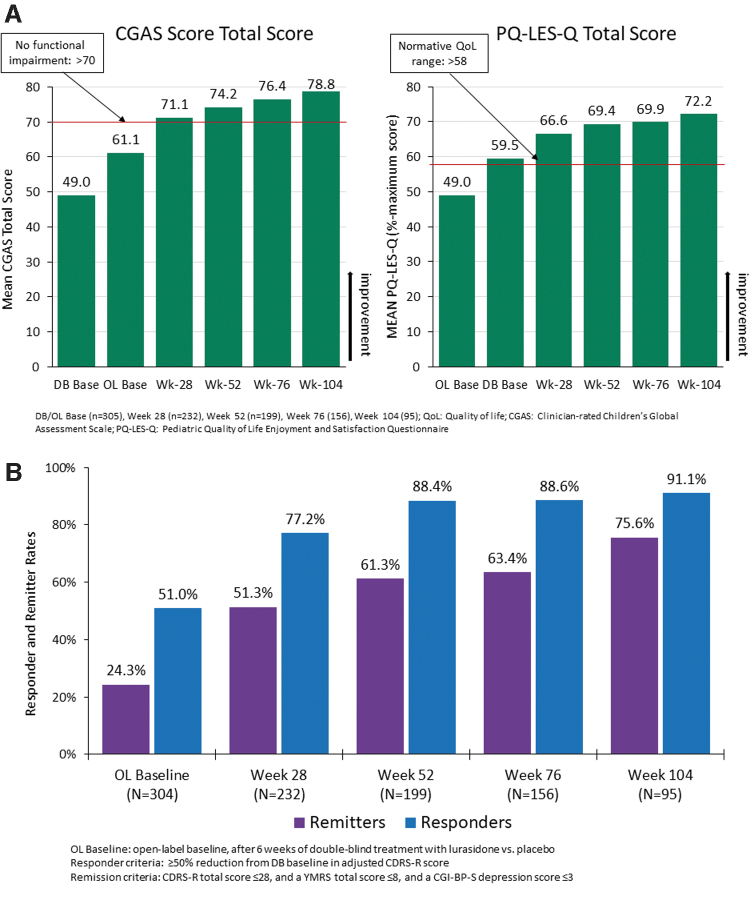
Secondary efficacy outcomes during OL treatment with lurasidone **(A)**. Change from DB baseline in CGAS and PQ-LES-Q scores OC, observed case analysis; **(B)**. Response and remission during OL treatment with lurasidone. CGAS, Children's Global Assessment Scale; DB, double-blind; OC, observed case analysis; OL, open-label; PQ-LES-Q, Pediatric Quality-of-Life Enjoyment and Satisfaction Questionnaire.

Functioning and quality of life, as measured by the CGAS and PQ-LES-Q, respectively, demonstrated progressive improvement across 104 weeks of lurasidone treatment, with the mean CGAS total score reaching normative levels of functioning at ∼52 weeks, and the mean PQ-LES-Q total score reaching normative quality of life levels by week 28 (Fig. 4**A**).

Responder rates (≥50% reduction from DB baseline in CDRS-R total score) increased during long-term treatment with lurasidone until reaching an asymptote of ∼85% to 90% at week 40 (Fig. 4B). The responder rate at week 104, based on a more conservative LOCF-endpoint analysis, was 81.7%. Remission (defined as subjects who had CDRS-R total score ≤28, YMRS total score ≤8, and CGI-BP-S depression score ≤3 at a given study visit) was achieved by ∼75% of participants at week 104 (Fig. 4B). The remission rate at week 104, based on a more conservative LOCF-endpoint analysis, was 61.7%. The Kaplan–Meier estimate of median time to first onset of remission was 86 days (95% CI: 85.0–197.0).

## Discussion

The results of this multicenter, OL extension study in youth (ages 10–17) with bipolar depression found lurasidone to be generally well tolerated during 2 years of long-term treatment, with a tolerability and safety profile consistent with results from previously reported short and long-term studies in children and adults with bipolar depression or schizophrenia (Loebel et al. 2014; Loebel et al. 2014a; DelBello et al. 2017; Citrome et al. 2012; Loebel et al. 2013; Loebel et al. 2015; Tandon et al. 2016; Goldman et al. 2017; Correll et al. 2020). The completion rate of 55% in the study was notably high and supports the tolerability of lurasidone over 2 years of treatment. No new or unexpected AEs were reported in the current study, and no deaths occurred. The attrition rate due to AEs was 7.8% at the end of 1-year, and 10.1% at the end of 2-years. Comparable prospective 2-year data are not available for other atypical antipsychotic drugs in an adolescent bipolar population. These results compare favorably to the one 12-month maintenance study we were able to find, where attrition due to AEs was12% in adolescents with bipolar disorder (57%) or schizophrenia (43%) treated with olanzapine (Detke et al., 2016).

Results from the structured UKU side effect rating scale were consistent with tolerability findings based on spontaneous AE reporting. Reductions were seen in AE severity across all UKU subscales along with an 80% overall reduction observed in the severity for the UKU side effect total score from OL baseline to LOCF-endpoint. The incidences of extrapyramidal symptoms and akathisia were low; and movement disorder assessments showed no clinically meaningful changes in EPS symptom severity. The low utilization rate for anticholinergic medication (5.2%) is consistent with this low reported risk of movement disorder effects from long-term treatment with lurasidone.

Consistent with results of a previously reported long-term lurasidone study in adolescents with schizophrenia (Correll et al. 2020), the present study found 2 years of treatment with lurasidone to have minimal effects on weight, lipids, and glycemic indices. This contrasts with the weight and metabolic effects reported in long-term (26-week) studies of olanzapine, the other USFDA-approved treatment for bipolar depression in youth. Specifically, treatment with olanzapine resulted in ≥7% weight gain in 55% and 69% of adolescents with bipolar disorder and schizophrenia, respectively, while shifts to abnormal lipid levels occurred in up to one-third of all participants (Tohen et al., 2007; Robertson-Plouch et al., 2007; McCormack et al., 2010).

The importance of metabolic and vascular safety as a major determinant of drug choice in the treatment of bipolar disorder, especially long-term therapy, has been insufficiently recognized until recently (Goldstein et al. 2015). A growing body of evidence indicates that bipolar disorder is associated with a vascular disease diathesis that consists of the following characteristics: increased risk of early obesity, increased risk of dyslipidemia, insulin resistance, early onset of metabolic syndrome, and the presence inflammatory and oxidative stress markers associated with vascular endothelial damage. These pathophysiological correlates of bipolar disorder in adolescents have been observed as white matter hyperintensities on brain magnetic resonance imaging (MRI) (odds ratio, 5.7 in bipolar adolescents vs. age-matched controls), and early onset of atherosclerotic changes. The adult consequence of this pathophysiology is a significant increased risk of vascular morbidity and mortality resulting in an average reduction in life expectancy of 10–15 years (Westman et al. 2013).

Continued reduction in depression symptom severity was observed during long-term lurasidone treatment across efficacy measures, including the CDRS-R total score and the CCI-BP-S overall and depression scores. Improvement in depression symptom severity was substantial, with the magnitude of improvement that occurred during OL treatment being comparable to what was observed during the initial 6-week DB treatment in the lurasidone group. The magnitude of improvement during extension phase treatment did not appear to be simply attributable to attrition bias for several reasons. First, only 1.6% of participants discontinued the study due to lack of efficacy. Second, summary results over time between study completers (i.e., observed cases) and all study participants using an LOCF approach were comparable. Third, comparable results were obtained using a sensitivity analysis based on multiple imputations of missing data.

Improvement in depression symptom severity during long-term treatment with lurasidone, while maintaining low manic symptomatology, resulted in bipolar remission rates that progressively increased over time, increasing to 76% in patients who completed 2 years of treatment. Normative levels of functioning also progressively increased, although as expected, functional improvement lagged several months behind improvement in mood symptoms.

Several important study limitations should be noted. Eligibility for enrollment in the DB trial was based on stringent study entry criteria that may have reduced the generalizability of the results. This was an OL trial that was not randomized or blinded and had no placebo or active comparator. Consistent with the long-term nature of the trial, concomitant medications were permitted, which may have reduced the incidence and/or severity of reported AEs. This is most notable for anxiety, which is a common part of the clinical presentation of bipolar depression. In the current study, treatment-emergent anxiety was reported by 8.5% of patients, while benzodiazepine use (some of which may have been prescribed for EPS or akathisia) was 16.7%. Finally, future long-term studies are needed to determine the efficacy of lurasidone in preventing recurrence of depression and mania, ideally utilizing a randomized withdrawal design, with DB, placebo substitution after various drug stabilization intervals.

## Conclusion

Up to 2 years of treatment with lurasidone in youth presenting with bipolar depression was generally safe and well tolerated, with a relatively low rate of study discontinuation, minimal impact on weight, metabolic parameters, and prolactin, and long-term improvement in depressive symptoms. Sustained improvement in affective symptoms was associated with return to normal levels of functioning and quality of life in the great majority of participants. Future controlled studies of lurasidone as a maintenance treatment for youth with bipolar disorder are needed.

## Clinical Significance

Due to the chronic and recurrent course of bipolar disorder in youth, long-term pharmacotherapy is typically required, making safety and tolerability crucial factors when determining the choice medications for this vulnerable population. However, few prospective, long-term studies are available on the safety, tolerability, and effectiveness of antipsychotic agents in this patient group. We have summarized here the results of prospective study demonstrating that up to 2 years of treatment with lurasidone in youth presenting with bipolar depression was generally safe and well tolerated, with a relatively low rate of study discontinuation, minimal impact on weight, metabolic parameters, and prolactin, and long-term improvement in depressive symptoms.

## Supplementary Material

Supplemental data

## Supplementary Material

Supplementary Appendix SA
