# Supplementary material for: Tolerability, Safety, and Effectiveness of Two Years of Treatment with Lurasidone in Children and Adolescents with Bipolar Depression
Source: J Child Adolesc Psychopharmacol. 2021 Sep 17;31(7):494–503. doi: 10.1089/cap.2021.0040 (PMC8568779; doi:10.1089/cap.2021.0040)
Supplement: Supplemental data [file Supp_AppS1.pdf]

Sensitivity Analyses testing the robustness of the CDRS-R effectiveness analyses

A. CDRS-R Total Score: Mean Change from Double-Blind Baseline by Dropout Visit Category

Completers vs. 3-month Dropout, 6-month Dropout, 1-year Dropout, Late Dropout) for Subjects Continued from the initial double-blind, placebo-controlled study (Safety, Observed)

Definition of dropout sub-categories:

- 3-month dropout: dropout on or prior to Week 12.
- 6-month dropout: dropout after Week 12 but on or prior to Week 28.
- 1-year dropout: dropout after Week 28 but on or prior to Week 52.
- Late dropout: dropout after Week 52 but prior to Week 104.
- Completers: subjects completed 104-week treatment.

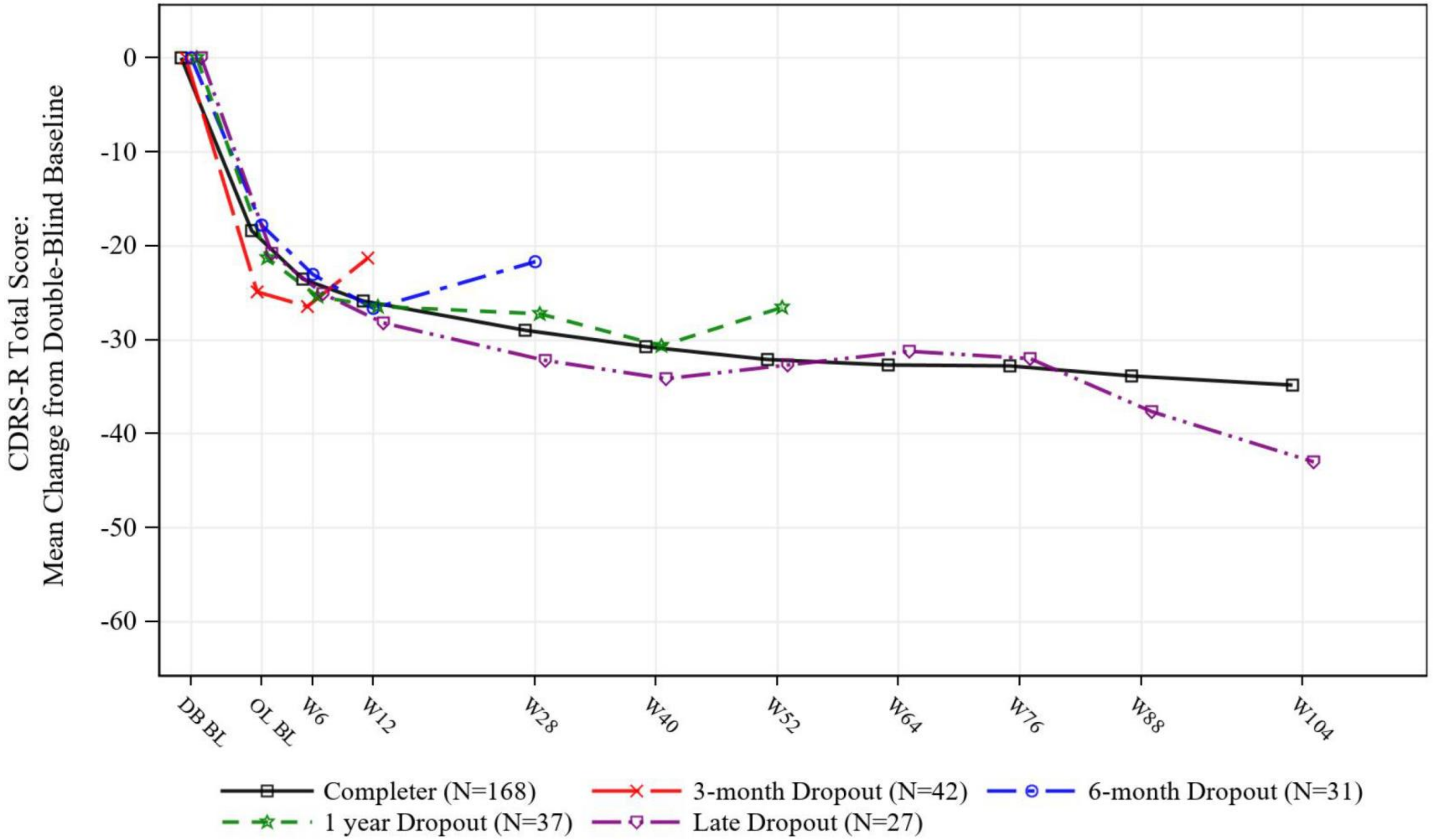

B. CDRS-R Total Score: Mean Change from Double-Blind Baseline by Dropout Reason Category for Subject Continued from Study D1050326 (Safety,
Observed)

Definition of dropout reason sub-categories:

- Adverse Event.
- Lack of Efficacy.
- Lost to Follow-up or Withdrawal of Consent.
- Protocol Violation or Other.
- Completers: subjects completed 104-week treatment.

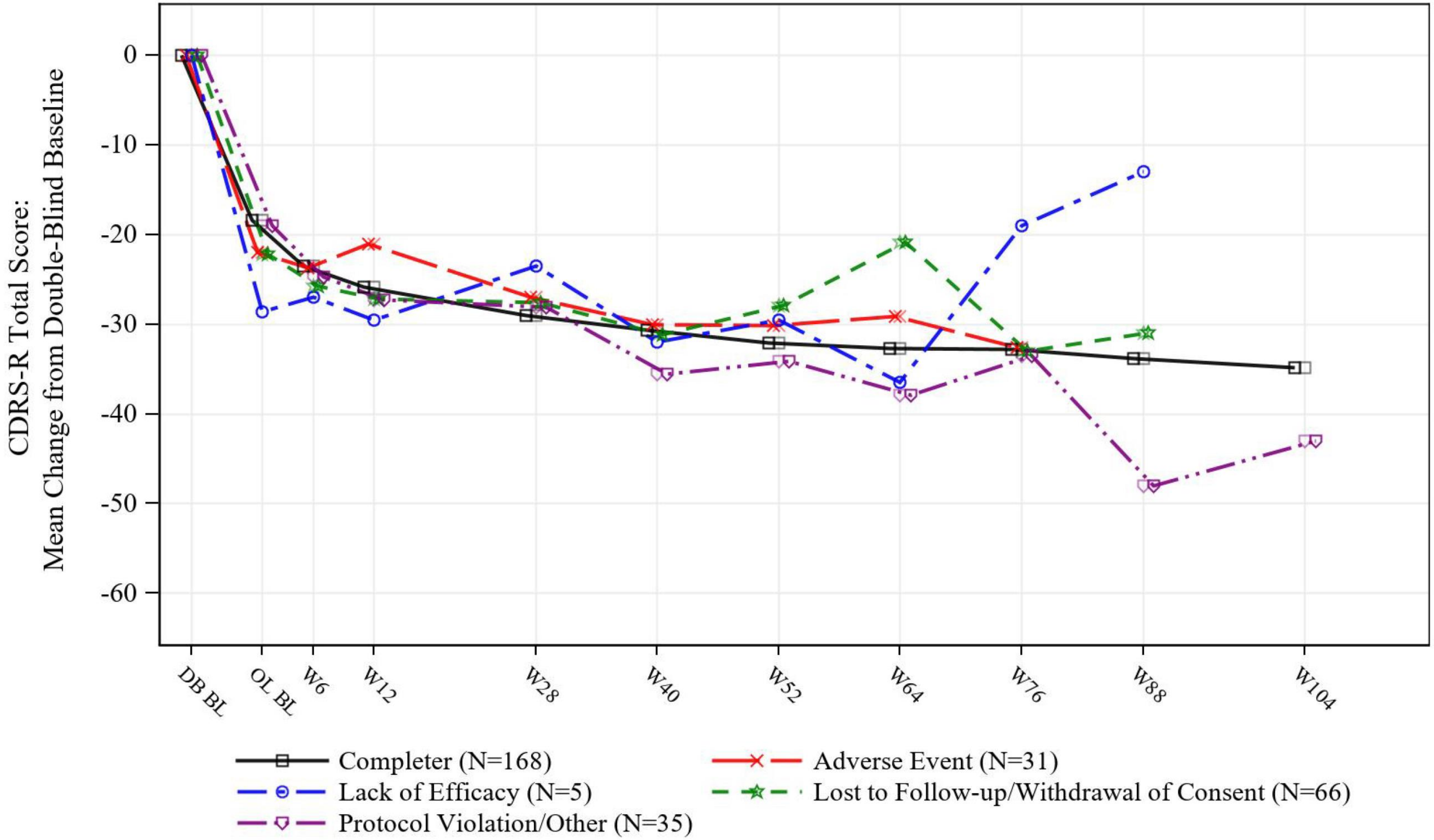

C. Multiple Imputation Approach for CDRS-R Total Score

Missing data of CDRS-R Total Score after dropout was imputed using multiple imputation methodology for subjects continued from the initial double-blind, placebo-controlled study. Baseline factors of 'pooled country' and 'age stratum' (ie, 10-14 years vs. 15-17 years at DB screening) as well as treatment assignment in the initial study (ie, lurasidone vs. placebo) were used in the multiple imputation process. Details of the procedure for implementation of the multiple imputations are as follows:

1. 1000 datasets were generated where missing data at intermediate visit(s) were imputed for all subjects using non-missing data from all subjects by a Monte Carlo Markov Chain (MCMC) imputation model using the MCMC statement in the SAS PROC MI procedure. As a result, each dataset only had missing ending data, or a monotone missing data pattern.
2. For each dataset from Step 1, missing ending data was imputed. As a result, 1000 imputed complete datasets were generated.
   - Missing data at the first post-open-label baseline visit was imputed by a regression imputation model using the SAS PROC MI procedure with the REGRESSION option in the MONOTONE statement.
   - The SAS PROC MI procedure used data from all subjects with missing data at the visit (i.e., only those that need imputation at the visit).
   - This was repeated for all other visits sequentially. Subjects whose missing data were imputed at previous visits contributed to the imputation for the next visit.
   - The regression imputation model included an intercept and the slopes of the measurements from all previous visits.
3. For each imputed complete dataset of CDRS-R Total Score:
   - Two types of variables were derived: change from DB Baseline and Change from OL Baseline
   - Change was analyzed from DB baseline and from OL baseline, respectively using MMRM model as below:
     The MMRM model included the following fixed terms: visit (as a categorical variable), pooled country, age stratum (ie. 10-14 years vs. 15-17 years at DB screening), CDRS-R Total Score at DB baseline, prior treatment (lurasidone vs. placebo), prior treatment*visit. The MMRM model with unstructured covariance was utilized for each imputed dataset.
     Timepoints in the MMRM model for change from DB baseline were: OL Baseline, W6, W12, W28, W40, W52, W64, W76, W88, W104.
     Timepoints in the MMRM model for change from OL baseline were: W6, W12, W28, W40, W52, W64, W76, W88, W104.
4. Estimates from the results of each MMRM model were combined
   - SAS MIANALYZE procedure was used to combine the MMRM results.

CDRS-R Total Score: Mean Change from DB Baseline over Time (Observed, LOCF vs Multiple Imputation) for Subject Continued from Study D1050326 (Safety)

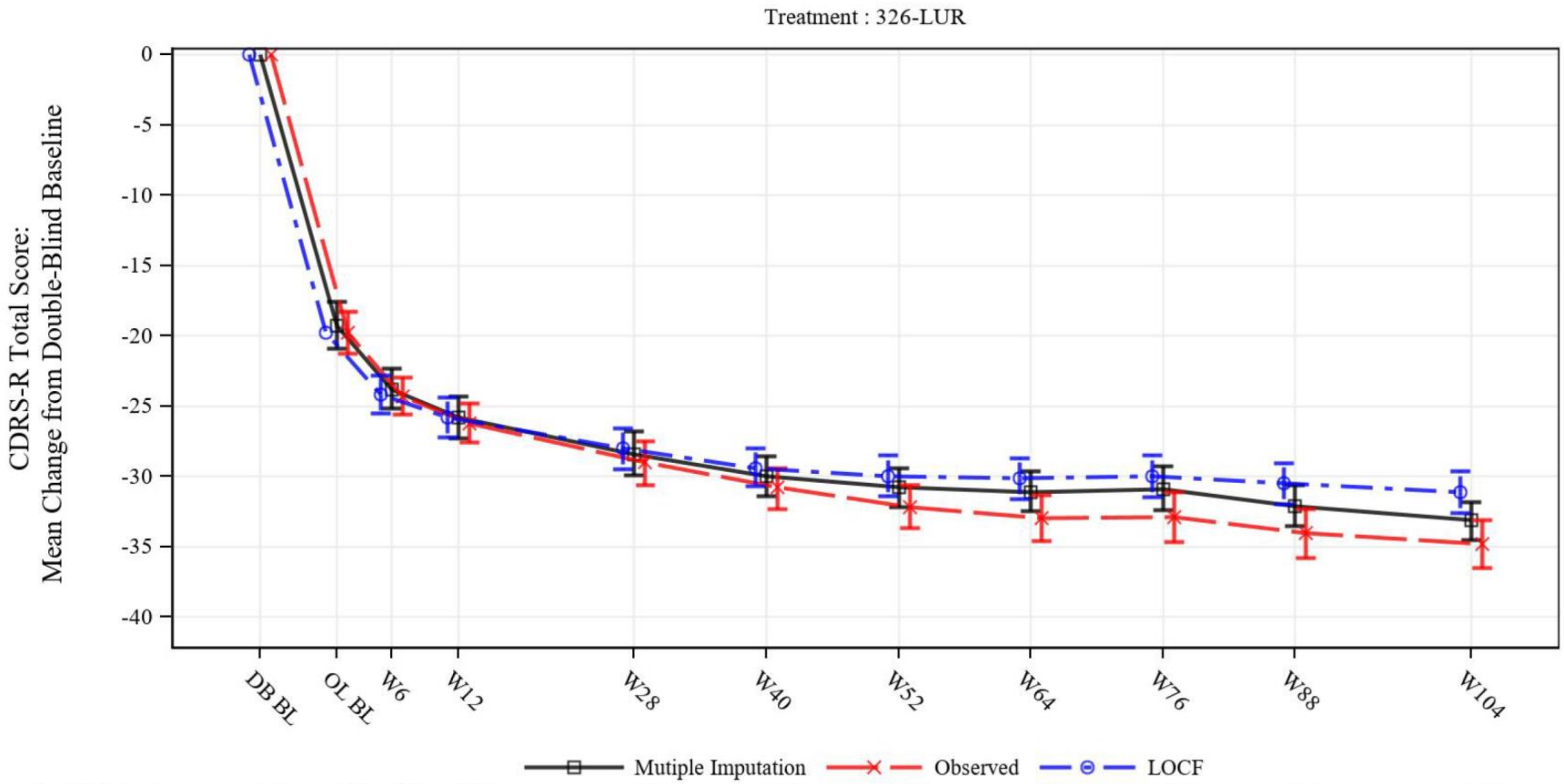

	DB BL	OL BL	W6	W12	W28	W40	W52	W64	W76	W88	W104
# of Subj: MI	305	305	305	305	305	305	305	305	305	305	305
# of Subj: Obs	305	304	282	266	232	218	199	188	175	171	168
# of Subj: LOCF	305	304	293	300	300	300	300	300	300	300	300

Note: LS means and their 95% CIs from the MMRM model are displayed for multiple imputation approach.
Abbreviation: MI= multiple imputation approach; Obs= observed case; LOCF = last observation carried forward.

CDRS-R Total Score: Mean Change from OL Baseline over Time (Observed, LOCF vs Multiple Imputation) for Subject Continued from Study D1050326 (Safety)

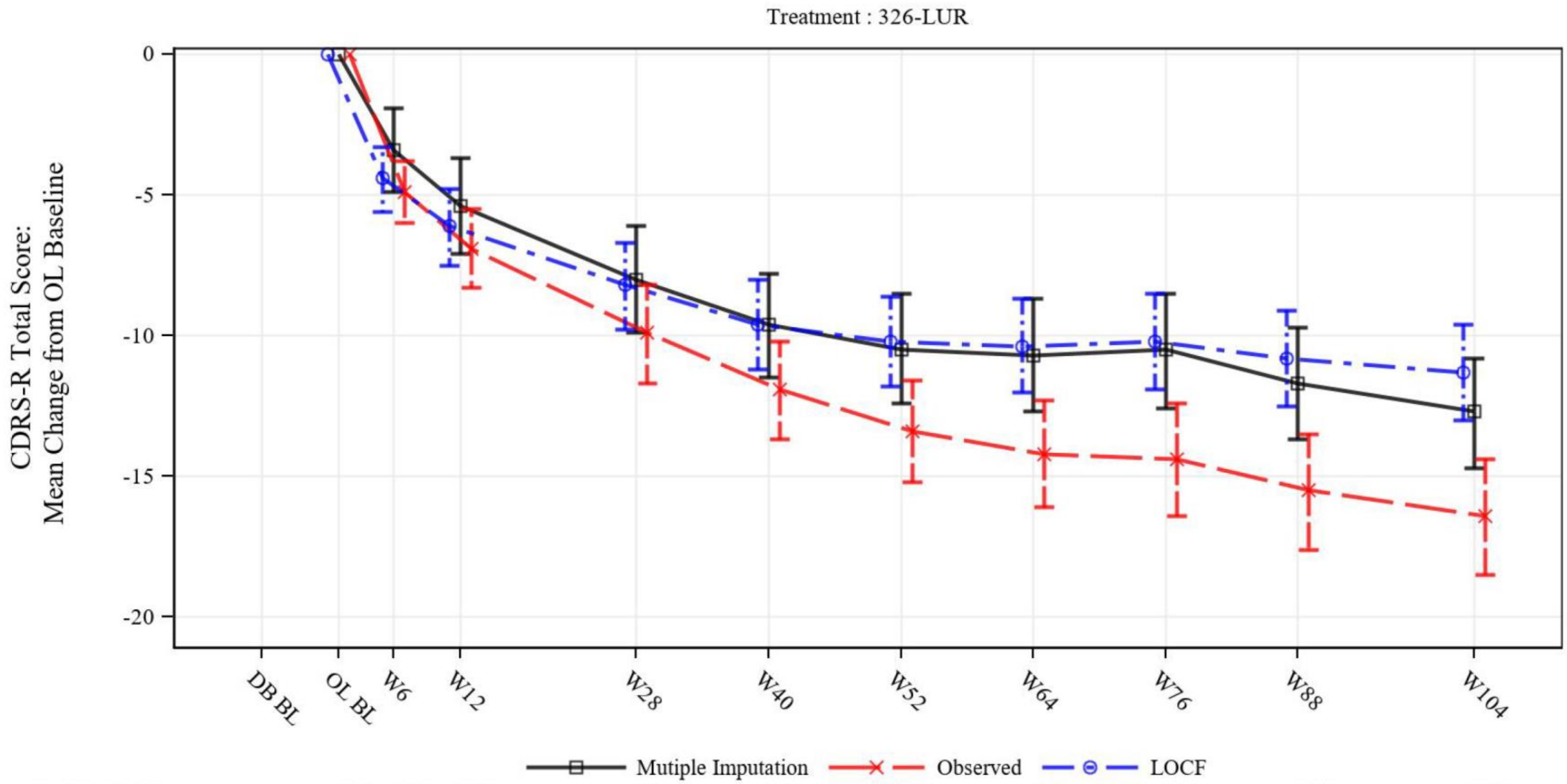

# of Subj: MI	305	305	305	305	305	305	305	305	305	305
# of Subj: Obs	305	281	265	231	217	199	188	175	171	168
# of Subj: LOCF	305	292	299	299	299	299	299	299	299	299

Note: LS means from the MMRM model are displayed for multiple imputation approach.
Abbreviation: MI= multiple imputation approach; Obs= observed case; LOCF = last observation carried forward.

Multiple Imputation Analysis of CDRS-R Total Score: Change from Baseline over Time - Mixed Model for Repeated Measures for Subject Continued from Study D1050326 (Safety)

Parameter/Visit	Statistics	PBO-LUR (N=149)	LUR-LUR (N=156)	326-LUR (N=305)
CDRS-R Total Score With Multiple Imputation				
Open-Label Baseline				
Change from DB Baseline	LS Mean (SE)	-16.6 (1.12)	-22.0 (1.09)	-19.3 (0.84)
	95% CI of LS Mean	(-18.8, -14.4)	(-24.1, -19.8)	(-20.9, -17.6)
Week 6				
Change from DB Baseline	LS Mean (SE)	-23.0 (0.97)	-24.6 (0.95)	-23.8 (0.75)
	95% CI of LS Mean	(-24.9, -21.1)	(-26.4, -22.7)	(-25.2, -22.3)
Change from OL Baseline	LS Mean (SE)	-5.3 (0.98)	-1.5 (0.95)	-3.4 (0.78)
	95% CI of LS Mean	(-7.2, -3.4)	(-3.3, 0.4)	(-4.9, -1.9)
Week 12				
Change from DB Baseline	LS Mean (SE)	-25.1 (1.00)	-26.4 (0.97)	-25.8 (0.76)
	95% CI of LS Mean	(-27.0, -23.1)	(-28.3, -24.5)	(-27.3, -24.3)
Change from OL Baseline	LS Mean (SE)	-7.4 (1.11)	-3.3 (1.07)	-5.4 (0.86)
	95% CI of LS Mean	(-9.6, -5.3)	(-5.4, -1.2)	(-7.1, -3.7)

Note: 1000 multiple imputations were performed using a monotone regression imputation method.
For each imputed dataset, MMRM (Mixed Model for Repeated Measures) was performed, based on the fixed effects for analysis group(PBO-Lur vs. Lur-Lur), pooled country (same as core study D1050326), age stratum (same as core study D1050326,ie, 10-14 years vs. 15-17 years), visit as a categorical variable, CDRS-R Total Score at DB Baseline, and analysis group by visit interaction, assuming an unstructured covariance matrix.

Multiple Imputation Analysis of CDRS-R Total Score: Change from Baseline over Time - Mixed Model for Repeated Measures for Subject Continued from Study D1050326 (Safety)

Parameter/Visit	Statistics	PBO-LUR (N=149)	LUR-LUR (N=156)	326-LUR (N=305)
Week 28				
Change from DB Baseline	LS Mean (SE)	-28.5 (1.05)	-28.3 (1.04)	-28.4 (0.80)
	95% CI of LS Mean	(-30.5, -26.4)	(-30.3, -26.3)	(-29.9, -26.8)
Change from OL Baseline	LS Mean (SE)	-10.8 (1.25)	-5.2 (1.23)	-8.0 (0.96)
	95% CI of LS Mean	(-13.3, -8.4)	(-7.6, -2.8)	(-9.9, -6.1)
Week 40				
Change from DB Baseline	LS Mean (SE)	-30.1 (0.92)	-30.0 (0.92)	-30.0 (0.72)
	95% CI of LS Mean	(-31.9, -28.3)	(-31.8, -28.2)	(-31.4, -28.6)
Change from OL Baseline	LS Mean (SE)	-12.4 (1.24)	-6.9 (1.23)	-9.6 (0.96)
	95% CI of LS Mean	(-14.9, -10.0)	(-9.3, -4.5)	(-11.5, -7.8)
Week 52				
Change from DB Baseline	LS Mean (SE)	-30.7 (0.90)	-30.9 (0.92)	-30.8 (0.72)
	95% CI of LS Mean	(-32.5, -29.0)	(-32.7, -29.1)	(-32.2, -29.4)
Change from OL Baseline	LS Mean (SE)	-13.1 (1.27)	-7.8 (1.26)	-10.5 (0.98)
	95% CI of LS Mean	(-15.6, -10.6)	(-10.3, -5.4)	(-12.4, -8.5)

Note: 1000 multiple imputations were performed using a monotone regression imputation method.
For each imputed dataset, MMRM (Mixed Model for Repeated Measures) was performed, based on the fixed effects for analysis group (PBO-Lur vs. Lur-Lur), pooled country (same as core study D1050326), age stratum (same as core study D1050326,ie, 10-14 years vs. 15-17 years), visit as a categorical variable, CDRS-R Total Score at DB Baseline, and analysis group by visit interaction, assuming an unstructured covariance matrix.

Multiple Imputation Analysis of CDRS-R Total Score: Change from Baseline over Time - Mixed Model for Repeated Measures for Subject Continued from Study D1050326 (Safety)

Parameter/Visit	Statistics	PBO-LUR (N=149)	LUR-LUR (N=156)	326-LUR (N=305)
Week 64				
Change from DB Baseline	LS Mean (SE)	-31.1 (0.96)	-31.0 (0.97)	-31.1 (0.76)
	95% CI of LS Mean	(-33.0, -29.2)	(-32.9, -29.1)	(-32.5, -29.6)
Change from OL Baseline	LS Mean (SE)	-13.5 (1.32)	-7.9 (1.30)	-10.7 (1.02)
	95% CI of LS Mean	(-16.1, -10.9)	(-10.4, -5.3)	(-12.7, -8.7)
Week 76				
Change from DB Baseline	LS Mean (SE)	-30.8 (1.01)	-31.0 (1.01)	-30.9 (0.79)
	95% CI of LS Mean	(-32.8, -28.8)	(-33.0, -29.0)	(-32.4, -29.3)
Change from OL Baseline	LS Mean (SE)	-13.1 (1.37)	-7.9 (1.35)	-10.5 (1.04)
	95% CI of LS Mean	(-15.8, -10.4)	(-10.5, -5.2)	(-12.6, -8.5)
Week 88				
Change from DB Baseline	LS Mean (SE)	-31.4 (0.94)	-32.7 (0.93)	-32.1 (0.73)
	95% CI of LS Mean	(-33.3, -29.6)	(-34.5, -30.9)	(-33.5, -30.6)
Change from OL Baseline	LS Mean (SE)	-13.8 (1.34)	-9.6 (1.31)	-11.7 (1.02)
	95% CI of LS Mean	(-16.4, -11.2)	(-12.2, -7.0)	(-13.7, -9.7)

Note: 1000 multiple imputations were performed using a monotone regression imputation method.
For each imputed dataset, MMRM (Mixed Model for Repeated Measures) was performed, based on the fixed effects for analysis group(PBO-Lur vs. Lur-Lur), pooled country (same as core study D1050326), age stratum (same as core study D1050326,ie, 10-14 years vs. 15-17 years), visit as a categorical variable, CDRS-R Total Score at DB Baseline, and analysis group by visit interaction, assuming an unstructured covariance matrix.

Multiple Imputation Analysis of CDRS-R Total Score: Change from Baseline over Time - Mixed Model for Repeated Measures for Subject Continued from Study D1050326 (Safety)

Parameter/Visit	Statistics	PBO-LUR (N=149)	LUR-LUR (N=156)	326-LUR (N=305)
Week 104				
Change from DB Baseline	LS Mean (SE)	-33.1 (0.87)	-33.1 (0.86)	-33.1 (0.69)
	95% CI of LS Mean	(-34.8, -31.4)	(-34.8, -31.4)	(-34.5, -31.8)
Change from OL Baseline	LS Mean (SE)	-15.5 (1.28)	-10.0 (1.24)	-12.7 (0.98)
	95% CI of LS Mean	(-18.0, -13.0)	(-12.5, -7.6)	(-14.7, -10.8)

Note: 1000 multiple imputations were performed using a monotone regression imputation method.
For each imputed dataset, MMRM (Mixed Model for Repeated Measures) was performed, based on the fixed effects for analysis group(PBO-Lur vs. Lur-Lur), pooled country (same as core study D1050326), age stratum (same as core study D1050326,ie, 10-14 years vs. 15-17 years), visit as a categorical variable, CDRS-R Total Score at DB Baseline, and analysis group by visit interaction, assuming an unstructured covariance matrix.
